# Mainstreaming Underutilized Indigenous and Traditional Crops into Food Systems: A South African Perspective

**DOI:** 10.3390/su11010172

**Published:** 2018-12-31

**Authors:** Tafadzwanashe Mabhaudhi, Tendai Polite Chibarabada, Vimbayi Grace Petrova Chimonyo, Vongai Gillian Murugani, Laura Maureen Pereira, Nafiisa Sobratee, Laurencia Govender, Rob Slotow, Albert Thembinkosi Modi

**Affiliations:** 1 Centre for Transformative Agricultural and Food Systems, School of Agricultural, Earth and Environmental Sciences, University of KwaZulu-Natal, P. Bag X01, Scottsville 3209, Pietermaritzburg, South Africa; 2 Soil, Crop and Climate Sciences, University of the Free State P.O Box 339, Bloemfontein 9300, South Africa; 3 Plant Soil and Microbial Sciences Department, Michigan State University, 1066 Bogue St A286, East Lansing, MI 48824, USA; 4 School of Life Sciences, University of KwaZulu-Natal, P. Bag X01, Scottsville 3209, Pietermaritzburg, South Africa; 5 Centre for Food Policy, City University of London, Northampton Square, London EC1V 0HB, UK; 6 Department of Genetics, Evolution & Environment, University College, London WC1E 6BT, UK

**Keywords:** agro-ecology biodiversity, climate resilience, health

## Abstract

Business as usual or transformative change? While the global agro-industrial food system is credited with increasing food production, availability and accessibility, it is also credited with giving birth to ‘new’ challenges such as malnutrition, biodiversity loss, and environmental degradation. We reviewed the potential of underutilized indigenous and traditional crops to bring about a transformative change to South Africa’s food system. South Africa has a dichotomous food system, characterized by a distinct, dominant agro-industrial, and, alternative, informal food system. This dichotomous food system has inadvertently undermined the development of smallholder producers. While the dominant agro-industrial food system has led to improvements in food supply, it has also resulted in significant trade-offs with agro-biodiversity, dietary diversity, environmental sustainability, and socio-economic stability, especially amongst the rural poor. This challenges South Africa’s ability to deliver on sustainable and healthy food systems under environmental change. The review proposes a transdisciplinary approach to mainstreaming underutilized indigenous and traditional crops into the food system, which offers real opportunities for developing a sustainable and healthy food system, while, at the same time, achieving societal goals such as employment creation, wellbeing, and environmental sustainability. This process can be initiated by researchers translating existing evidence for informing policy-makers. Similarly, policy-makers need to acknowledge the divergence in the existing policies, and bring about policy convergence in pursuit of a food system which includes smallholder famers, and where underutilized indigenous and traditional crops are mainstreamed into the South African food system.

## 1 Introduction

Agriculture became the backbone of food systems more than 10,000 years ago, as humans shifted from hunting and gathering to growing and cultivating food [1]. It has since been instrumental to rising civilizations and their growing populations, and, over time, has evolved as humans have acquired more knowledge and innovation. The Industrial Revolution, which started in Europe in the 1800s, was an important event characterized by an improvement of farming practices and the invention of new tools [1]. Following this, there was an occupational shift as people left farming to work in the factories, cities mushroomed, a strong low to middle class emerged, and economic and income growth occurred. This necessitated a shift in the food system to allow greater production to supply the labor force in cities [2].

By the mid-1900s, world population was ≈ 2.5 billion. Whereas the industrialized countries were largely food secure, countries in Latin America, Asia and Africa continued to suffer from chronic hunger [3]. The Green Revolution led to development of high-yielding varieties of major cereal crops [maize (*Zea mays*), wheat (*Triticum aestivum*), rice (*Oryza sativa*)] that were responsive to additional inputs such as fertilizers and water, resulting in the birth of an agro-industrial food regime [3,4]. To an extent, Asian and Latin American countries benefited from the Green Revolution, saving them from famine [3,5]; however, this impacted smallholder agriculture with a shift towards greater dependency on the agro-industrial food system [6,7]. Inevitably, the successes of the Green Revolution and subsequent emergence of the agro-industrial food system gave birth to ‘new’ challenges, such as environmental pollution and degradation [3,5], loss of biodiversity [5,8], and malnutrition [9]. What is noticeable is that sub-Saharan Africa (SSA) was left behind in the first Green Revolution, and there is now a controversial, but concerted effort to enable a ‘new’ green revolution for Africa [10,11].

In spite of the technological advances, the global food system is failing to meet the basic food needs of the world’s citizens equitably. These challenges currently dominate the global Sustainable Development Agenda. Additional technological improvements towards the end of the 20th century, combined with a consolidation of capital in the food system to a few multi-national corporations, further entrenched the agro-industrial food system into what McMichael [12] refers to as the ‘corporate food regime’. The current global food system remains a diverse mixture of localized and industrialized systems of interconnected food chains [2,13–15]; however, the majority of these systems are centered on a handful of crop choices [16]. As a result, modern food systems are more vulnerable to economic and climatic shocks, as they may not always have the requisite diversity and redundancy to be able to buffer these risks—i.e., they are not resilient [17]. This is especially true in developing economies that are currently experiencing climate variability and change [18].

A lesser documented outcome of the global agro-industrial food system has been the post-colonial replacement, and subsequent relegation, of underutilized indigenous and traditional crops through the introduction of exotic and, now considered “major” crops, that were often higher yielding, but also more input intensive [19,20]. This led to neglect of underutilized indigenous and traditional crop species that had previously formed the basis of local food systems, especially in the global South. The displacement of indigenous and traditional crop species by a few major crops has inevitably contributed, in part, to the limited successes of the global food systems, especially in underdeveloped regions of the world [21]. This is also evident by the minimal influence exerted by smallholder rural farmers, also referred to as family farmers, who are custodians of underutilized indigenous and traditional crops. While the global agro-industrial food system has recognized the role played by smallholder rural farming systems [22], these groups of farmers marginally influence the system, and are at great risk from economic and climatic shocks [23]. This is because they have limited access to the modernized inputs, techniques, and markets necessary to participate in the production of major crops [24,25]. The erosion of agro-biodiversity, combined with an emphasis on input-intensive cropping systems has, arguably, lowered the resilience of food systems in the global South [26]. In this paper we have adopted the South African definition for underutilized indigenous and traditional crops as described by Modi and Mabhaudhi [27]. They defined underutilized indigenous and traditional crops as “crops that have either originated in South Africa or those that have become “indigenized” over many years (>10 decades) of cultivation as well as natural and farmer selection within South Africa.” The term ‘indigenous’ has also often been used to refer to crops that may have originated elsewhere but have undergone extensive domestication locally, thus giving rise to local variations, i.e., ‘naturalized/indigenized crops’ [28]. Indigenized crops are sometimes referred to as traditional crops [28]. For examples of underutilized indigenous and traditional crops, the reader is referred to Chivenge et al. [29]. Underutilized indigenous and traditional crops are often characterized by limited development relative to their potential. Consequently, they have poorly developed and understood value chains; however, this varies across geographic and socio-economic settings.

South Africa is one of the few African countries that has been embedded in the global agro-industrial food system for decades [30]. Despite this consolidation, South African food prices remain too high for the majority of her people, who, consequently, cannot afford to purchase adequate food, leaving 21.3% of the population with poor access to food [31]. Furthermore, the concerns regarding environmental degradation, loss of biodiversity, and vulnerability to climate change, have prompted a call to rethink the current configuration of the South African food system [32]. A focus on reinvigorating underutilized indigenous and traditional crops, and bringing these to the market, has been suggested as an entry point for improving diets and making them more sustainable [32–34].

In this regard, we reviewed the status of South Africa’s food system with the aim to identify opportunities for mainstreaming underutilized indigenous and traditional crops into the food system. The specific objectives were to review the current status of the food system and its limitations, and to identify opportunities for mainstreaming indigenous crops for environmental sustainability and improved health outcomes, resilient agricultural systems, and agro-ecological biodiversity. Within the context of this review, the term “mainstreaming” is used to refer to the integration/inclusion of underutilized indigenous and traditional crops into the dominant food system. However, such integration or inclusion should occur in a way that allows them to retain the attributes that make them attractive and transformative while benefiting from the support mechanisms that exist within the dominant food system.

## 2 Methodology

### 2.1 Literature Review

The review followed the PRISMA guideline (www.prisma-statement.org) for a structured review. A mixed-method review approach, which included combining quantitative and qualitative research, was used to compile the review. Scientific journal articles and book chapters were obtained from databases such as JStor^®^, Scopus, ScienceDirect^®^, and Cab Direct^®^, while technical reports and other forms of literature were obtained from Google™ and South African Government Gazettes. The main search key terms included “Food systems in South Africa”, “Indigenous crops and Food Systems”, “Traditional crops and Food Systems”, “Agro-ecology biodiversity and Food Systems”, “Food Systems and Health”, “Food system and Environment”. Search terms were set to be in the title, keywords, or abstract. Results from all the databases totaled 13,145 papers (Figure S1), and were exported to Mendeley desktop (Elsevier, USA), where duplicates were immediately removed. Further to this, false hits, publications in languages other than English, and articles that had only abstracts available were also removed. Following this, there were 276 articles remaining (Figure S1). The literature was then subjected to review by relevant experts who further filtered the papers, and also added peer reviewed literature relevant for the review that was not obtained in the literature search (Figure S1). Although the review has a particular focus on South Africa, literature and case studies from outside South Africa, especially developing countries, were obtained, and also used as examples in the review. Eventually 127 articles consisting of scientific research, working papers, Government Gazettes, and popular articles, were used to compile the review. The review was then structured into three sections; the current status of food systems in South Africa (Section 3), opportunities for underutilized indigenous and traditional crops into a new food systems paradigm (Section 4), and, lastly, the study recommendations (Section 5).

### 2.2 A Systemic Analysis of the Opportunities for Underutilized Indigenous and Traditional Crops in the Current SA Food System

As a means to gain a ‘real world’ perspective on the ways in which the food system can be leveraged to create an enabling environment for the empowerment of historically underprivileged farmer communities and the inclusion of underutilized indigenous and traditional crops in smallholder farming, qualitative system dynamics models were used to show the inter-linkages in the system. The Vensim PLE x32 software (Ventana Systems Inc., Harvard MA, USA) was used to construct the system maps, which combines causal loop diagramming (CLD) and stock accumulation in a qualitative portrayal of the dynamics of the food system. In the CLD, arrows show the influence of one variable on another—a change in the cause leads to a change in the effect. The polarity of the arrows indicates the factual relationship between any two nodes, which illustrates the causal link. Simple stock and flow networks are also used to depict accumulation, and the corresponding rate of change over time. In trying to understand a particular ‘system of interest’, the interplay of balancing and reinforcing loops gives rise to a realistic multi-loop system that explains behavior through time [35]. In the present paper, the system of interest refers to the mainstreaming of underutilized indigenous and traditional crops into the food system in post-apartheid South Africa.

## 3 The Current State of Food Systems and Underutilized Indigenous and Traditional Crops in South Africa

Overall, the current status of the food system in South Africa remains a reflection of the legacy of apartheid policies. Land ownership and, therefore, crop production for commercial consumption was largely the preserve of white commercial farmers, while smallholder farmers’ produce was largely for own-consumption and sale in informal markets [36]. The result is a dichotomous food system where the commercial sector supplies the dominant food system while the smallholder producers channel their produce to household consumption and alternative food systems that are poorly developed. Consequently, smallholder producers and low-income households also have to rely on the dominant food system [37,38]. Despite reference in agricultural transformation policies to improve smallholder farming that is still practiced by previously disadvantaged South African smallholder farmers, the food system remains largely untransformed. The objective of this section was to assess the current status of South Africa’s food system and its limitations, and to identify opportunities for mainstreaming underutilized indigenous and traditional crops with regards to food and nutrition security, agriculture and economic exclusion, environment and policy.

### 3.1 Food and Nutrition Security

Food and nutrition security is the ability to obtain safe, nutritious foods to meet the basic dietary requirements of an individual, in order to perform daily duties [39], while malnutrition refers to deficiencies (undernutrition), excesses (overnutrition), or imbalances in a person’s intake of nutrients [40]. Although the current food system has the capacity to feed the South African population [41], food remains inaccessible to ≈ 26% of the population [31]. Approximately 16 million people are food insecure, and the trends show that there has been no evidence of a decline in these numbers, and the majority of the food insecure population in South Africa is in rural areas [42]. Recent statistics on malnutrition show that 43% of children under five are malnourished [stunting (27%), wasting (3%) and overweight (13%)]. In addition to this, 68% of women in South Africa are overweight [43]. This highlights the flaws in the food systems and the country’s nutrition agenda. Over-nutrition has been linked to several non-communicable diseases such as diabetes, hypertension and cardiovascular disease [9,44].

**Figure 1:**
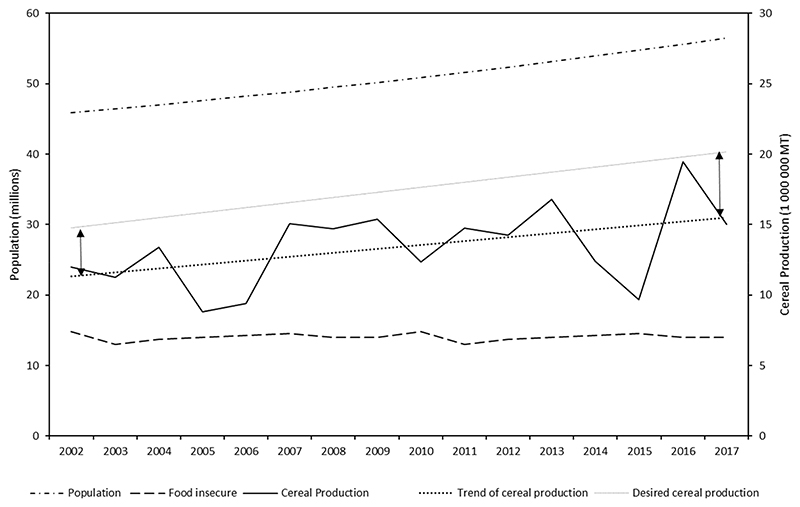
Population in South Africa, population of food insecure and the current and desired trend in food production (Figure developed by authors using population and food insecu rity data from the General Household Survey [31] and cereal production data from www.grainsa.co.za.

While there is evidence that food production is increasing, this is not enough to feed the growing population and the food insecure (Figure 1). To feed the growing population, and to close the food insecurity gap, food production has to increase by at least 30% of the current production (Figure 1). The gap between achieved food production and the desired food production seems to be increasing compared to previous decades (Figure 1). While agriculture is the main source of livelihood for poor rural households [31,45], it is under marginal conditions, and often cannot sustain subsistence [29,46]. In addition, these households have limited buying power and cannot cope with high food prices [8]. Current agricultural policies have been structured to promote production of cash crops such as maize, wheat, and sugarcane (*Saccharum officinarum*) no enable them to sell the surplus after own consumption [37,38]. These crops are energy dense, and, if consumed alone, do not provide adequate nutrition and lack micronutrients [47]. This is all evident in the high prevalence of all forms of malnutrition seen in young children and women in South Africa [42]. In this regard, underutilized indigenous and traditional crops could be an alternative to bridge the food and nutrition security gap, especially in the rural areas. Several underutilized indigenous and traditional crops have been reported to be nutrient-dense with good adaptability to marginal conditions; hence, they are more likely to be a sustainable and nutritious source of food [29].

The Agricultural Policy in South Africa recognizes the role of smallholder farmers; however, it is silent on the issues of underutilized indigenous and traditional crops. This is consistent in other strategy documents issued by the Department of Agriculture [48]. Instead, these documents focus on developing an emerging class of smallholder farmers with commercial aspirations. Though not mentioned explicitly, it can be surmised that this would be through growing major crops, which fit into the dominant food system. These same priorities are reflected in the National Development Plan which prioritizes the commercialization of crops which are in line with the dominant food system [49]. The National Food and Nutrition Security Policy has similar priorities, as it speaks about efficient agricultural production, and is largely silent about the cultivation of underutilized indigenous and traditional crops and development of alternative food systems that include these crops [50]. These policies do, however, advocate for the consumption of nutrient-dense underutilized indigenous and traditional crops [50].

Although current policies reflect a developmental agenda and aim to include and develop small family farmers, they do not fully consider their limitations and opportunities for inclusion into the dominant food systems. Such inconsistencies may be a symptom of the divergent views held by policy-makers on how to address problems of equity, food and nutrition security, and poverty reduction. These different views may be influenced by the success past government policy played in supporting commercial farmers, so that South Africa would be food secure [51]. An unintended consequence has been the resulting skewness of household food insecurity, as income poor rural households who should access food through self-production, purchase most of their food from markets [34]. This is consistent with views of policy-makers worldwide, who perceive the inclusion of small farmers in food systems as a means to improve rural livelihoods, but who are torn between including them in the dominant food systems or creating alternative food systems for them [52]. There is space for underutilized indigenous and traditional crops in this discourse, however, they are often excluded from policy and strategic documents, as shown in their exclusion from The Plant Improvement Act [53].

**Figure 2:**
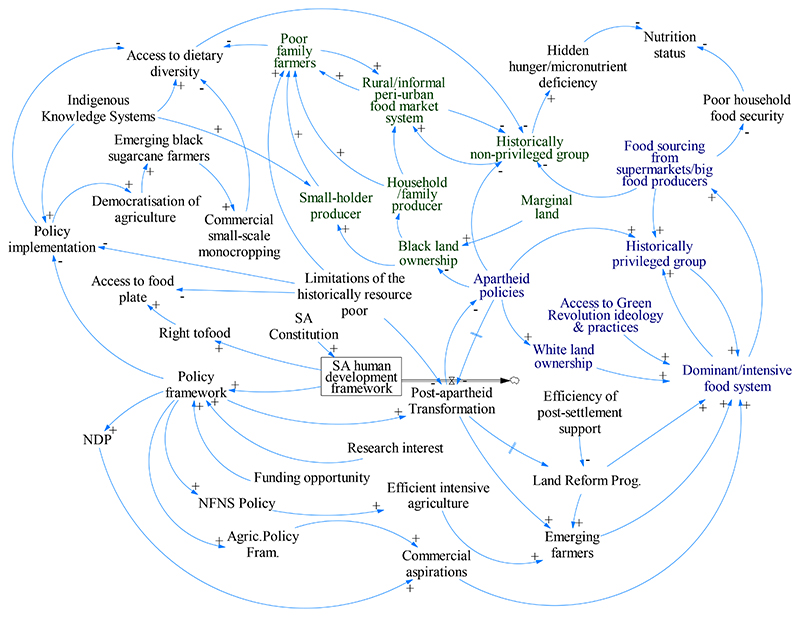
System map depicting the current dichotomous nature of the food system in South Africa. Nodes in blue and green color indicate the dominant and the smallholder producer food systems, respectively. The black nodes represent the opportunities and limitations of the current policy landscape in enabling the smallholder communities to create and sustain a viable food system. An ‘equal’ sign on an arrow denote systemic delay in the causal relationship. Agric. Policy Fram.: Agricultural Policy Framework; Land Reform Prog.: Land Reform Programme; NDP: National Development Plan; NFNS: National Food and Nutrition Security.

What has emerged from our systems analysis (Figure 2), is that current policy frameworks have inevitably resulted in dual outcomes of increased “hidden hunger” and reduced poor household food and nutrition security, leading to increased malnutrition and all of its associated consequences (Figure 2). Key factors leading to this emergent outcome are Apartheid policies, policy framework, dominant intensive food system, and the pervasive limitations faced by poor family farmers to be food secure (Figure 2). A counterintuitive effect of the post-apartheid policy implementation has been the democratization of agriculture that enabled underprivileged smallholder producers to embark on sugarcane (*Saccharum officinarum*) monocropping. However, this shift decreased food crop production and dietary diversity. All of these realities would need to be addressed to counter the vicious cycle to malnutrition, as depicted at the top right of Figure 2. The recognition of the importance of Indigenous Knowledge Systems, for instance, through a formalization of the indigenous knowledge base, could be used as an entry point to leverage the incorporation of underutilized indigenous and traditional crops within policy implementation processes, in order to improve access to dietary diversity among the previously disadvantaged. This group is currently caught in a vicious circle of malnutrition, which is exacerbated by poor socio-economic outcomes for household food and nutrition security, and an unsustainable reliance on big food producers and the informal peri-urban food market system. The various consequences of malnutrition for human wellbeing are exacerbated by their inherent vulnerability in the socio-economic system.

### 3.2 Agriculture and Economic Exclusion

Current agricultural activities recognize the need for increased crop productivity to fight poverty, unemployment and food and nutrition insecurity [48]. However, many of them remain modelled on green revolution ideology, that emphasizes efficiency and productivity over resilience [54]. In South Africa, current policies, funding opportunities, and research interests are still trying to push yield potential of a few major crops, and are not geared towards the development of an indigenous food crop sector. This has made the promotion of underutilized indigenous and traditional crops within current crop and food systems challenging. It also explains the apparent low biodiversity in the country’s agro-industrial system. There is growing evidence that shows linkages between low agro-biodiversity and the failures of current food systems to deliver adequate quantities of healthy, nutritionally balanced food, especially to underprivileged people [34,44,55,56]. The global focus on a few crops has resulted in reduced nutrition, which has become more conspicuous in rural areas [57]. Diversity of diet, founded on diverse farming systems, delivers better nutrition and greater health, with additional benefits for human productivity, livelihoods and wellbeing [58,59]. Agricultural biodiversity will be essential to cope with malnutrition, and to establish more sustainable food systems. The inclusion of underutilized indigenous and traditional crops and associated alternative food systems into policy frameworks can result in improvements of agro-biodiversity.

The importance of smallholder farmers to food systems, and their participation in local food systems, must be emphasized. The large-scale commercial farming sector dominates production of agricultural commodities, both for the ‘formal’ and ‘informal’ segments of the agro-food system in South Africa [60]. Given the dominance of large-scale and corporate farming business, the mainstream agricultural supply chains are failing to distribute enough food for the country on their own [34]. A paradigm shift is required for the future of farming and food systems in South Africa if government is to deliver on its promises of food and nutrition security for all. A dynamic approach is needed, moving away from a rigid industrial approach; putting smallholder producers at the center of food systems. Extensive evidence suggests smallholder farming systems, also referred to as family farming systems, can help feed a growing South African population [61]. Their participation requires unblocking ideological barriers biased in favor of industrial agriculture; understanding the ways to facilitate and augment agro-ecological practices, local, and traditional knowledge systems; and, re-orientating and prioritizing of local agro-food systems [62]. Supporting local food chains for underutilized indigenous and traditional crops is vital to improve local demand, and improve opportunities for smallholder farmers to increase participation in national and regional food chains. Ultimately, supporting local agricultural systems will improve the interest of farmers in cultivating underutilized indigenous and traditional crops, which have the potential to be profitable cash crops [63,64].

The inequality reflected in the production system is mirrored in consumption practices. Socio-economic factors contribute significantly to what one purchases from the food system for household consumption [65]. Yet, what is offered by the current food system in many countries is often not representative of a nation’s cross-section of cultural and religious dietary requirements [66]. This affects both producers and consumers. The exclusion of underutilized indigenous and traditional crops from the dominant food system, likely due to poorly developed value chains, disadvantages smallholder farmers, particularly female farmers who are responsible for the conservation, production, and processing of underutilized indigenous and traditional crops [67]. The establishment of such value chains would be beneficial to those households who reside in urban and peri-urban areas, where vegetables and other fresh produce are scarce and therefore expensive [68]. Consumers belonging to minority and marginalized groups, who have little buying power, often find that the dominant food system does not offer what they require [69]. These consumers often turn to informal food systems to purchase their food choices, which includes underutilized indigenous and traditional crops that are often not available from the dominant food system [68].

### 3.3 Environment

The ongoing intensification of agricultural production in South Africa has had particularly notable effects on the environment through release of greenhouse gases, pollution, loss in species biodiversity and erosion. In South Africa, it is estimated that agriculture contributes ≈12% of global anthropogenic greenhouse gas (GHG) emissions, releasing 21,714 GgCO_2_eq in 2010 [70]. In general, agricultural production, including indirect emissions associated with land use changes and direct emissions from land clearing, contributes 80%–86% of total food system emissions while processing, transporting, storing, cooking and disposing of food contributes the remaining 14%–20% [58,71]. There were no statistics to separate GHG emissions of smallholder and commercial agriculture. However, it is hypothesized that commercial agriculture could possibly contribute to more GHG emissions due to its reliance on external inputs and energy to drive machinery [70]. Animal production contributes a bigger carbon and water footprint compared to plant production. This higher carbon footprint is associated with their feeding, processing and the release of methane gas by ruminants such as cows (*Bos Taurus*) [58,72]. Per ton of product, animal sourced foods have up to a 20 times larger water footprint than crop products [73]. In South Africa, there is an increase in the consumption of animal sourced foods as socio-economic status improves and the replacement of traditional diets by more Westernized diets occurs [74]; hence an increase in health and environmental burdens. The environmental benefits of reducing the fraction of animal sourced foods in food systems are known [75]. Transitioning toward more plant-based food systems could reduce food-related greenhouse gas emissions by 29%–70%[75]. In support of this notion and to further diversify current plant-based food systems, we advocate for the inclusion of nutrient-dense underutilized indigenous and traditional crops [50].

Other concerns are the contribution of agriculture to fine particulate matter in the form of ammonia emissions from animal production and manure processing activities, and, to a lesser extent, fertilizer use [76]. Ammonia emissions have an atmospheric lifespan which ranges from days to weeks, and pollute whole regions in the process, affecting both ecosystems and human health [77–79]. In addition, the application of agrochemicals and fertilizer to increase yield in the dominant food system is associated with possible contamination of soil and water through the wrong application or over-usage of these chemicals [80,81]. Uncontrolled application of pesticides can kill other non-target and beneficial organisms such as bacteria, fungi, and earthworms. Microbial biomass is a labile component of soil organic matter and has an important role in the soil nutrient element cycle [82]. Agrochemicals can move from agricultural fields into nearby streams, rivers and lakes where their toxicity could pose a risk to aquatic ecosystems. These agrochemicals can vary significantly in their toxicity towards aquatic organisms as well as their mobility in the environment—properties which are influenced by their chemical make-up and other climatic, geographic and land management factors. Nitrogen and phosphorus release from agricultural fields also results in eutrophication of aquatic ecosystems, leading to the loss of biodiversity, imbalance of species distribution, shifts in the structure of food chains, and impairment of fisheries [83]. Within avian populations found in the country, 11 species are listed as critically endangered and 43 species as vulnerable [75]. The sensitivity of avian species to agrochemical pollutants has also been widely reported [75]. There is need to moderate and regulate the use of agrochemicals in agriculture to reduce the impacts of food systems to the environment. Underutilized indigenous and traditional crops are less susceptible to pests and diseases and require less fertilizer interventions; hence, they can mitigate the negative environmental impacts of agrochemicals.

While underutilized indigenous and traditional crops may offer some reprieve to environmental issues, there is a need to complement efforts to mainstream them into the dominant food system with sound agricultural practices for the system as a whole. This would ensure current impacts of agriculture on environmental degradation are minimized and food systems become more sustainable. Additional considerations in this regard include, but are not limited to, the use of sustainable and climate smart agriculture techniques that speak to adaptation, mitigation and sustainable intensification of production systems.

### 3.4 Policy

The policies governing the food system reflect a favorable environment for big businesses, and have made it conducive for a few players to dominate the food system [84]. Many policy-makers favor the commercial agenda because its actors have shown that it is productive; improving national food security, reducing unemployment, and contributing to the national gross domestic product (GDP) [68,85]. However, the profit-making aspects of this food system, which make it attractive, also expose its cost in the form of environmental harm, and inequitable distribution, which results in household food and nutrition insecurity [52,86]. The inability of the dominant food system to distribute produced food adequately to ensure household food security is glaring [68,87]. Thus, two types of policy gaps are revealed, on the one hand policies with divergent goals, and, secondly, policy gaps at critical points of the food system, which have largely been left to big business to address.

It is evident that the status quo is unsustainable. The food system is under pressure to achieve equitable distribution of food produced in the food system, and to feed the growing population using the resources already dedicated to agriculture [85]. Arguments have been made for the inclusion of underutilized indigenous and traditional crops in the existing food system. The first is that there is potential to increase crop diversity and thereby to increase dietary diversity, thus achieving food and nutrition security outcomes [5]. Secondly, advocates for an inclusive food system propose the strengthening of local food systems [88]. This move is seen as setting communities and nations on the path to achieving food sovereignty, which is seen as a key ingredient to achieving both national and household food and nutrition security [85]. These arguments show that there is room for underutilized indigenous and traditional crops in the current food system, however, their inclusion cannot happen effectively in the current policy environment.

It can be argued that policies which seek to enhance smallholder farmer participation in the dominant food system may also expand the inclusion of underutilized indigenous and traditional crops in the food system. However, this is not so, as often when smallholder farmers participate in activities in the dominant food system, they do so by adopting its few crops [67]. These include either staple crops like hybrid maize, or cash crops like sugarcane (*Saccharum officinarum*) or exotic vegetables such as peppers (*Capsicum spp*.) that are more water intensive than alternative traditional crops. The extension system exacerbates these trends, as it is not designed to promote the kind of knowledge that is required to invigorate traditional farming practices that are conducive to cultivating underutilized indigenous and traditional crops.

The South African government amended the National Health Act in 2003, as part of its mandate to ensure basic health services were available to everyone. As part of its mandate, it aims to educate people on healthy food, monitor dietary patterns, nutrient intakes, and nutrition status indicators to promote human health and to prevent diseases [89]. However, because the South African food system is largely driven by market forces, these are not always aligned with nutrition goals. Inorganically processed food has been a major component of diets in South Africa, and has been associated with overweight in both children and adults, as well as chronic diseases such as cancer [90]. This gave rise to a growing market for organic food, which is often very expensive and is beyond the reach of the majority of the population. Ironically, rural farming systems closely resemble organic farming as a result of minimal use of synthetic inputs; however, while their production systems may resemble organic farming, for farming systems to be considered organic, they need certification, which this group of farmers cannot afford [91]. In addition, the extension system is designed to modernize their production systems and increase the use of inorganic inputs like pesticides and fertilizers [92,93].

There is a clear need for a shift in the policy environment, one that recognizes that the ‘business as usual’ attitude, and piece-meal policy inclusions, will not result in the improved presence of underutilized indigenous and traditional crops in the food system; unless they are intentionally included in the food system [52]. This demands that policy-makers recognize that transformative approaches are required, and that this will require follow-through and not mere rhetoric. This process can be initiated by policy-makers acknowledging the divergence in the existing policies and taking it as an opportunity to bring about policy convergence in pursuit of an inclusive food system. Getting underutilized indigenous and traditional crops onto the policy-makers’ agenda could lead to their inclusion in future policies; however, advocates for such progressive ideas often lack the power to do so [84]. There may be need for an alliance with actors with more power to influence agenda setting in the policy arena. Once such items are on the policy agenda, policy-makers can also direct the research agenda to fill the research gap on underutilized indigenous and traditional crops’ breeding, processing etc. [94,95]. However, merely getting underutilized indigenous and traditional crops onto the agenda is not enough, the resulting policies should be implemented to bring about inclusivity and equity in the food system, and to strengthen its contribution to the local economy and the GDP. South Africa has had a draft Indigenous Food Crops Strategy in the works since April 2014, but as yet, nothing has come of it.

In addition, government needs to develop policies to address areas in the food system which they may have previously perceived as outside their purview, e.g., the distribution and marketing of food. The policy-makers need to adopt a transformative approach, which embraces informal traders and transporters, who, while already active in the food systems, operate illegally [68]. These informal traders and transporters are an important conduit for including underutilized indigenous and traditional crops in the current system, and, thus, their activities must not be stifled, but, rather, must be incorporated into the dominant food system. Embracing these informal traders and creating a policy environment which makes it conducive for them to distribute food, including underutilized indigenous and traditional crops, to poor communities, will prevent the proliferation of food shortages in underserviced communities.

## 4 Way Forward for Diverse and Indigenous Food Systems

**Figure 3:**
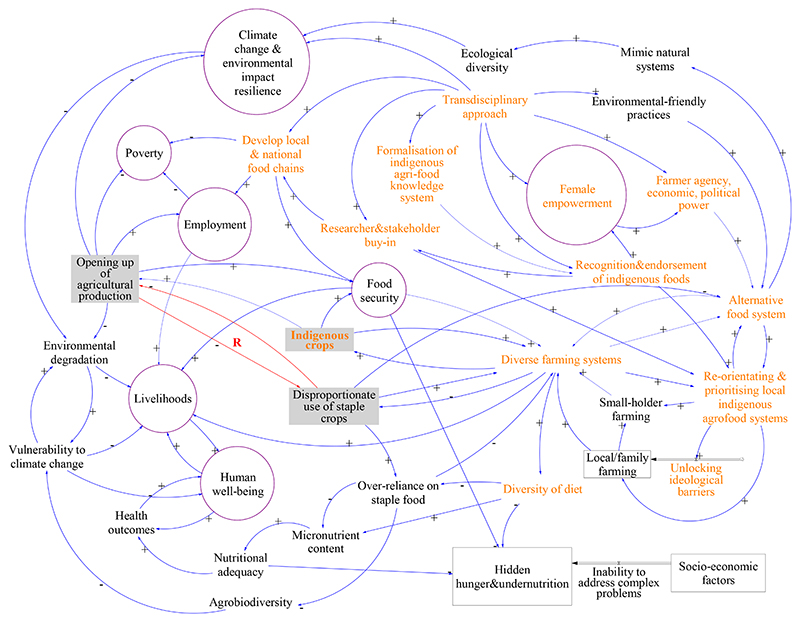
Leveraging thee current SA food system for the resource poor. The orange nodes denote thee interventions that expand beyond the parochial, to change the system towards sustainability. Variables with a gray background reveal the important nexus points of the system. Encircled nodes in purple color demonstrate how the some of the subcomponents of the system converge towards achieving specific SDG relevance. By opening up diverse agricultural production, the current state which is chara cterized by dispropo rtionate use of stap le crops could b s counteracSed within a virtuous cycle (denoted by the red arrows) that promote diversified crop production.

Given the evidence presented above, in what ways can the paradigm shift be steered to support vulnerable populations, and improve their wellbeing? What type of evidence should be created to support policy-makers in creating the opportunities for supporting smallholder farming? Local/family farming is a subset of the smallholder system, which itself is embedded in the wider socio-economic and agro-ecological systems (Figure 3). The quality of the food plate of vulnerable populations can be improved through the introduction, or re-introduction, of the underutilized indigenous and traditional crops into the food systems. In order to create an enabling environment for the change to happen at the local level, a multi-disciplinary approach, combining research and practitioner-led interventions, is proposed to inform policy makers of the landscape that needs to be created (Figure 3).

### 4.1 Addressing Health and Nutrition through Diversity

The current food system in South Africa has been shown to lack dietary diversity and has exposed ≈26% of the population to food and nutrition insecurity (c.f. Secticn 3.2). Young children and women of child-bearing age in rural areas are often most vulnerable, as they lack access to a diversified diet which leads to malnutrition [42]. On a global scale, there are approximately 7000 known and documented edible species of plants [96]; however, due to globalization, there is a decline in the consumption of underutilized indigenous and traditional crops [97]. With climate change, and the fluctuation in food prices [96], it becomes very important to cupport traditional crops and farming systems [97]. Currently, traditional farming systems are poorly developed and not well-markcted [97]. Including underutilized indigenous and traditional crops into a new food system will increase dietary diversity. Several underutilized indigenous and traditional crops, especially vegetables, have high nutritional value, and could improve the nutritional status of many impoverished individuals [95].

African leafy vegetables such as amaranth (*Amatanthus* spp.) Chinese cabbage (*Brassica rapa*), black nightshade (*Solanum nigrum*), Jew’s mallow (*Corchorus olitorius*), cowpea leaves (*Vigna unguiculata*), pumpkin leaves (*Cucurbita spp*.), tsamma melon (*Citrullus lanatus*), and spider flower (*Cleome gynandra*) have been reported to be good sources of vitamins, fiber and iron [98]. These vegetables have also been reported to be used in other parts of South Africa but have been facing marginalization due to low economic value [99,100]. These crops could contribute to alleviating malnutrition in South Africa [98]. Cowpea and bambara groundnut (*Vigna subterranea*) are other examples of underutilized indigenous and traditional crops that are gaining popularity [101]. Although crops such as bambara groundnut are gaining popularity, not many consumers are familiar with the production and preparation of this crop, which may pose a challenge to uptake [101]. By incorporating underutilized indigenous and traditional crops into the food system there will be an increase in dietary diversity, improvement in nutritional status, and a reduction in household food and nutrition insecurity [102]. However, basic knowledge on the production and preparation of underutilized indigenous and traditional crops should be provided to farmers and impoverished individuals, to improve acceptance. When underutilized indigenous and traditional crops are incorporated into a new food system, there should be a transdisciplinary approach, so that new technologies can be used to add value to the products [103].

### 4.2 Addressing Socio-Economic and Environmental Concerns through Agro-Biodiversity

The consumption of underutilized indigenous and traditional crops in many communities in South Africa and worldwide was adversely affected by their perception as ‘poverty foods’ or food for the elderly [100,104]. Colonization and globalization contributed to the introduction of foods which were considered ‘modern’ and, therefore, more attractive than traditional foods [100]. However, some of these ‘modern’ foods were not as nutritious as underutilized indigenous and traditional crops, and, subsequently, narrowed dietary diversity [88]. In addition, the consumption of underutilized indigenous and traditional crops declined as they were not included in the dominant food systems and could only be purchased in alternative and informal food systems [68]. These alternative food systems, however, were also slowly displaced with the proliferation of regional supermarket chains in both urban and rural South Africa [68].

The agro-industrial food system has inadvertently disempowered farmers, particularly smallholder farmers, many of whom are female [71]. Thus, any attempts to include underutilized indigenous and traditional crops in the food system should, ideally, begin with imparting skills which will increase farmer agency, and economic and political power [71,84]. Such initiatives would not only equip farmers to demand services and opportunities, but would position them as equal partners in the exchange of knowledge between themselves and researchers and other stakeholders [84]. This is important because elderly farmers, who are custodians of underutilized indigenous and traditional crops’ conservation and knowledge in most communities [100], are needed to document the different species of underutilized indigenous and traditional crops and their uses. Publishing and recording the information on locally available underutilized indigenous and traditional crops and their uses has been associated with an increase in the use of underutilized indigenous and traditional crops in local diets [88], which created demand for underutilized indigenous and traditional crops in the beneficiary communities [88,105]. The endorsing of underutilized indigenous and traditional crops by influential persons and bodies can also improve their demand in the food system, thus creating opportunities for inclusion in the value chain [88]. This has been observed in some southern African countries, which are battling with both malnutrition and the high incidence of non-communicable diseases [100]. Medical endorsements of underutilized indigenous and traditional crops, which are positioned as healthy and nutrient-rich foods [100], have led to increased consumption, particularly by wealthier members of society [95]. Mainstreaming into the diet of wealthier people would, presumably, also make these crops more attractive and aspirational to poor households, thereby overcoming any previous stigma (see above). Such a surge in consumption of underutilized indigenous and traditional crops can potentially reduce malnutrition and the prevalence of non-communicable diseases, while simultaneously stimulating market opportunities for farmers [85,95].

Cultivating underutilized indigenous and traditional crops in an inclusive food system could contribute significantly to addressing environmental concerns. Agro-ecological practices and other farming systems which mimic nature would be instrumental in reducing impacts [85]. By their nature, such farming systems promote the growth of a multiplicity of edible and medicinal plants which are indigenous to a region [67,84,85]. Such crops, which had been relegated to alternative food systems, and are therefore not widely available, can once again be introduced and integrated into the dominant food system. Increasing their prominence in the food system would also serve as a means of fostering their conservation, as these species would otherwise be lost through underutilization and land use changes [88,106]. Furthermore, a system which is based on species diversity is resilient and can withstand different threats and shocks, climatic or otherwise [16,107].

This sustainability extends to agricultural livelihoods in the event of an environmental shock, as some crops would survive these events, and, thus, reduce farmer vulnerability [16]. Such benefits would be appealing to farmers who are increasingly at risk of being affected by climate change events, but who have no access to insurance [108]. The added benefit of growing underutilized indigenous and traditional crops is that they provide dietary diversity, which has a lower carbon footprint than animal sources [58]. Indigenous species are agroecologically adapted to their local environment, and often grow spontaneously with few added inputs, which significantly reduces the pollution of soil and water from the introduction of agricultural chemicals [88]. Furthermore, when the cultivation of underutilized indigenous and traditional crops is coupled with little land disturbance, this would then reduce the extent to which farmers disturb the ecosystem, reducing environmental degradation.

The environmental benefits of including underutilized indigenous and traditional crops in the food system notwithstanding, a significant constraint in the adoption of these strategies is the diminishing knowledge surrounding their uses, and perceptions of low productivity [84,109]. Documenting and popularizing the benefits of planting underutilized indigenous and traditional crops, for instance legumes as part of crop rotations or intercrop systems, could appeal to both environmentally conscious and resource poor farmers [110]. For those farmers who have concerns about the productivity of farming systems which mimic nature, research is showing that, when well-managed, they perform better than monoculture farming systems, which are common in the dominant food system [85]. The resulting crop diversity and high yields may be an adequate incentive for those who wish to adopt sustainable agricultural practices on a large scale, but had previously found them not to be financially rewarding [107]. Thus, incorporating underutilized indigenous and traditional crops into the production system will result in improved sustainability and resilience in the food system [102].

Figure 3 elicits and captures the transdisciplinary approach to re-orient and prioritize local indigenous agro-food systems, namely: (i) the creation of an alternative and diverse food system that empowers local smallholder famers; (ii) that caters to the nutritional health and livelihood of the resource poor; and, (iii) that addresses climate and environmental change impacts and builds resilience. The influence of including a transdisciplinary approach, recognizing/endorsing underutilized indigenous and traditional crops, developing local food chains, resulting in diverse farming systems and alternative food systems, would create a virtuous cycle of increased household food and nutrition security, improved livelihoods, and reduced malnutrition (Figure 3), thereby reducing vulnerability, inequalities, and improving human wellbeing.

### 4.3 Agriculture-Environment-Health Nexus

Based on the review on the current status of the South African food system (c.f. Section 3) there was scant evidence of an appreciation of the linkages between agriculture, environment, and health in the current agro-industrial food system. The three sectors need to work together in order to address common issues such as improved agricultural productivity, food and nutritional security, reduced environmental degradation, improved human health outcomes, and improved human wellbeing in general. It is clear that agriculture is responsible for increasing food production and influencing healthy diets. Consumers are important players in the food system and their demands can influence production, as shown by the increased demand for animal sourced foods in countries experiencing rapid economic growth [111]. It is possible that improving consumer education on the link between certain foods and their carbon footprint can reduce the demand for those foods. There is evidence to show that reducing the consumption of beef results in lower carbon and ammonia emissions, despite increasing food demand [112].

**Figure 4:**
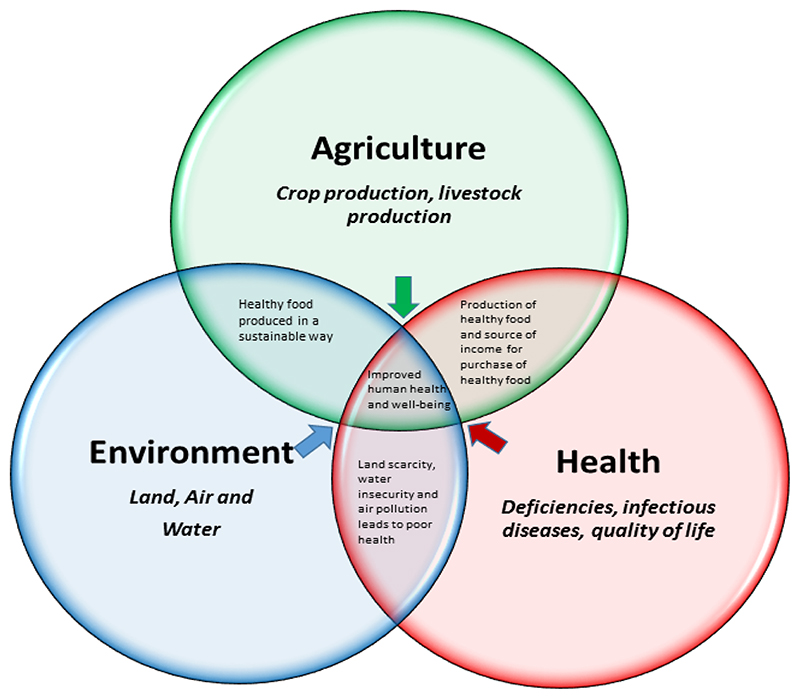
Agriculture-Environment-Health Nexus showing the interconnectedness and linkages between agriculture, environment and health sectors. The nexus outcome of improved human health and well-being is an outcome of a sustainable and healthy food system inclusive of underutilized indigenous and traditional crops.

However, agriculture is not just about growing food for consumption, there are also aspects of the environment that are key for agriculture [113,114]. Key inputs in agriculture include water and land, thus, any consideration of increasing food production will need to consider water and land use [113,114]. Given the challenges of water scarcity and associated challenges in expansive agriculture, there is a need for an agriculture-environment-health nexus approach to address the sustainable provision of enough nutritious food for supporting healthy diets (Figure 4).

The contribution of agriculture to food and nutrition security and, ultimately, human health, is not only through the direct provision of food. Benefits of agriculture can also be indirect, for example, agriculture is already a source of income for ≈65% of South African households [115]. In rural parts of South Africa, smallholder farming systems have been associated with women as their source of livelihood and employment [29]. Such income can be used by women to buy food that can improve food and nutrition security, and health. According to the Organization for Economic Co-operation and Development (OECD) agriculture plays an important role for poverty reduction and economic development in developing countries [116,117].

Conceptually, analyses of the agriculture-environment-health nexus require a food tystems approach that recognizes the interdependence and interconnectivity of these sectors. Furthermore, this approach emphasizes that the food system comprises a set of activities ranging from production to consumption, and that each of these activities, such as the processing of food, impacls all of these areas. Underutilized indigenous and traditional crops engender several of the key nexus points within the agriculture-environment-health nexus (Figure 4). Firstly, with regards to the agriculture-environment linkages, underutilized indigenous and traditional crops are often produced within niche agro-ecologies, and hence require less landscape modification [103]. This also supports the notion that they support environmentally sustainable and resilient agriculture [103]. Similarly, with regards to the agriculture-health linkages, the inclusion of underutilized indigenous and traditional crops offers prospects for broadening food and nutrition security (dietary diversity) [45] as well as income security for smallholder farmers [103] (c.f. Section 3.2). With respect to the environment-health linkages, as already established, the adaptation of most underutilized indigenous and traditional crops to harsh agro-ecologies and marginal production areas, means that they exert less pressure on already scarce land and water resources [29]. Lastly, owing to these attributes, underutilized indigenous and traditional crops have been reported to fit within the water-food-nutrition-health nexus as they can be used as part of efforts to improve human health and nutrition is water scarce environments [103].

## 5 Conclusions and Recommendations

A sustainable and healthy food system delivers food and nutrition security for all, in a way that is economically, socially, and environmentally sound, so as not to compromise food and nutrition security for future generations [118]. Underutilized indigenous and traditional crops can support and strengthen the existing food system, as they in particular are considered as economically, socially, and environmentally sound [28]. Several underutilized indigenous and traditional crops are nutrient dense and adapted to marginal conditions, suggesting that they could be used to champion sustainable and resilient agriculture and food systems for smallholder farmers residing in these environments [28].

The mainstreaming of underutilized indigenous and traditional crops should not seek to transform them into “new major crops” but should recognize their attributes that make them desirable as well as the role played by their current custodians in conserving them. In this regard, the fact that value chains for underutilized indigenous and traditional crops are currently poorly developed creates opportunities to diversify the current food systems, thus creating new employment, market, and distribution opportunities, and promoting autonomous pathways out of poverty. While the agro-industrial food system has inadvertently excluded smallholder farmers and women in rural areas, underutilized indigenous and traditional crops offer the opportunity for such groups of farmers to re-enter the system. Importantly, the significant role played by women in the production and conservation of underutilized indigenous and traditional crops [119] offers opportunities for women empowerment through their inclusion in the food system. Promoting gender equality and women’s empowerment is inextricably linked to the strengthening of sustainable food systems to fight hunger and malnutrition, and improving the lives and livelihoods of rural populations. The mainstreaming of underutilized indigenous and traditional crops into the food system would support women to diversify their landscapes in a sustainable way, feed their own households, and to provide nutritious food at local markets. In this regard, promotion and inclusion (i.e., mainstreaming) of underutilized indigenous and traditional crops could contribute towards addressing Sustainable Development Goals related to social, economic, and environmental issues; specifically, SDGs 1, 2, 3, 8, and 15 [120].

It is acknowledged that smallholder farmers have low literacy and numeracy skills, and that this may hamper efficient production and the adoption of new technologies. Initiatives taken to integrate smallholder farmers into the agro-industrial food system require a strong training component to address underdeveloped skills [121]. There is a positive correlation between farmer training and technology adoption, and this has often resulted in positive economic and environmental outcomes [112,121]. This calls for all the role players, from researchers to policy makers, to view these sectors in a nexus approach, and not to view them as separate, siloed, sectors.

Our systems analysis revealed the importance of inequalities and power imbalances, especially as a legacy of Apartheid discriminative policies, resulting in reduction in diversity of crops, reduced access to the food system, and reduced diversity of diet; these have outcomes of reduced household food security and increased “hidden hunger”, both of which feed into malnutrition and all its consequences (Figure 3) in a vicious cycle. When we include underutilized indigenous and traditional crops into the food system, the increased diversity in crops, improved local value chain, and diversified food system, result in increased household food security, improved livelihoods, and reduced hidden hunger (Figure 3), a virtuous cycle.

Policy-makers need to transform policy-making processes to represent the interests of different food system actors, promote underutilized indigenous and traditional crops, and support systems at all stages of the food system. This is an important point about the policy-making process. As we advocate for transdisciplinary research, which involves the various actors’ knowledge co-creation and co-development, this approach should also be applied for policy making. It should be truly participatory, and provide for influence by beneficiaries as required by transformation, and by societies’ values, as per global human rights and the SA constitution. The current policy environment makes little mention of underutilized indigenous and traditional crops, and is thus unfavorable to actors who grow, process, and distribute underutilized indigenous and traditional crops in informal food systems. In addition, the current policy environment leaves critical aspects of the food system, such as marketing and distribution, to the private sector, which may have a different agenda. Furthermore, it leaves the holders of the indigenous and local knowledge about these diverse crops vulnerable to corporate patents that do not take into account access and benefit sharing legislation [122–125]. Transformative change should be underpinned by robust scientific evidence. Thus, the current body of research that has been generated on underutilized indigenous and traditional crops, as highlighted in this review, needs to be translated for uptake by policy-makers. This highlights a gap in terms of the role of researchers—that there is not effective translation of work to make it accessible and relevant to policy makers, or to policy processes. With the point above, this means that two key actors are missing in the policy process, the scientists (at the start of the policy value chain), and the beneficiaries (at the end of the value chain). Another element that is often ignored or marginalized is indigenous knowledge systems, which provide an alternative information/evidence source to western-driven science; indigenous knowledge also needs to be made accessible and translated for decision-makers [126].

The different power relations in the food system must be recognized, as these may influence the success of any interventions. Smallholder farmers who currently produce underutilized indigenous and traditional crops are disempowered compared to other food system players, because of their poverty, and gendered access to resources and opportunities, as they are predominantly female. There is a need to rebrand and reposition underutilized indigenous and traditional crops as an important component of a sustainable and healthy food system.

Our review highlights the unintended consequence of a commercialized food system, based on a reduced number of crops, as reinforcing inequality and imbalances. While increasing national food security and stimulating national GDP, rather than making food cheaper and more accessible to all members of society, such a food system creates imbalances, reduces household food and nutrition security, and exacerbates existing inequalities. Therefore, instead of improving the wellbeing of all, as envisaged in the Sustainable Development Goals, such a food system is disempowering, increases vulnerability of the most vulnerable, and perpetuates and creates legacy imbalances in outcomes such as health, wealth, and education, which will have long-term effects on national development and nation building. Such insights, explored in the South African context, have similar implications for other developing countries faced with competing policy agendas of increasing agricultural production for commercial growth and development, versus ensuring affordable and household food and nutrition security for the most vulnerable citizenry, thereby decreasing social cohesion, and increasing the threat of social instability and conflict. Future studies should focus on conducting an econometric assessment to determine a cost-benefit analysis for mainstreaming underutilized indigenous and traditional crops into the South African food system. This would go some way in informing policy of the value of such action.

## Supplementary Material

PRISMA Checklist

PRISMA Flowchart
