# Supplementary material for: Mainstreaming Underutilized Indigenous and Traditional Crops into Food Systems: A South African Perspective
Source: Sustainability. Author manuscript; Available in PMC 2023 Sep 7. (PMC7615043; doi:10.3390/su11010172)
Supplement: PRISMA Checklist [file EMS187259-supplement-PRISMA_Checklist.pdf]

PRISMA 2009 Checklist

Table S1. PRISMA 2009 Checklist.

Section/topic	#	Checklist item	Reported on page #
**TITLE**			
Title	1	Identify the report as a systematic review, meta-analysis, or both.	1
**ABSTRACT**			
Structured summary	2	Provide a structured summary including, as applicable: background; objectives; data sources; study eligibility criteria, participants, and interventions; study appraisal and synthesis methods; results; limitations; conclusions and implications of key findings; systematic review registration number.	1
**INTRODUCTION**			
Rationale	3	South Africa is one of the few African countries that has been embedded in the global agro-industrial food system for decades. Despite this consolidation, South African food cost remains too high for people to afford, leaving 21.3% of the population with poor access to food. Furthermore, the concerns on environmental degradation, loss of biodiversity, and vulnerability to the impact of climate change, have prompted a call to rethink the current configuration of the South African food system. A focus on reinvigorating indigenous crops and bringing these to the market has been suggested as an entry point for improving diets and making them more sustainable.	1
Objectives	4	The specific objectives were to review the current status of the food system and its limitations and to identify opportunities for mainstreaming indigenous crops for improved health, environment, agricultural production and agro-ecology biodiversity.	3
**METHODS**			

Protocol and registration	5	PRISMA 2009, www.prisma-statement.org	2
Eligibility criteria	6	Studies reporting on Food systems, indigenous crops and aspects of the review from any year were selected. Only publications in English and full text were considered.	2
Information sources	7	Scientific journal articles and book chapters were obtained from databases such as JStor®, Scopus®, ScienceDirect®, and Cab Direct® while technical reports and other forms of literature were obtained from Google™ and South African Government Gazettes	2
Search	8	The main search key terms included “Food systems in South Africa”, “Indigenous crops and Food Systems”, “Traditional crops and Food Systems”, “Agro-ecology biodiversity and Food Systems”, ‘Food Systems and Health”, “Food system and Environment”. Search terms were set to be in the title, keywords and abstract.	2
Study selection	9	Based on relevance to the review	2
Data collection process	10	Subject expertise conducted quality assurance on the data,	2
Data items	11		
Risk of bias in individual studies	12	Where studies resorted on contrasting views it is indicated in the review.	2
Summary measures	13	Data from Grain South Africa was used to draw Figure 1.	2
Synthesis of results	14	A mixed-method review approach, which included combining quantitative and qualitative research, was used to compile the review	2
Risk of bias across studies	15	N/A as review as a mix of qualitative and quantitative.	
Additional analyses	16	Data from Grain South Africa was used to draw Figure 1 while other statistics is reported as given in the literature.	
**RESULTS**			

Study selection	17	There was no statistical analyses.	
Study characteristics	18		
Risk of bias within studies	19		
Results of individual studies	20		
Synthesis of results	21		
Risk of bias across studies	22		
Additional analysis	23		
**DISCUSSION**			
Summary of evidence	24	Our systems analysis revealed the importance of inequalities and power imbalances, especially as a legacy of Apartheid discriminative policies, resulting in reduction in diversity of crops, reducing reduced in access to the food system, and reduced diversity of diet, which has an outcome of reduced household food security and, increased "hidden hunger", both of which feed into malnutrition and all its consequences, a vicious cycle. When we include indigenous crops into the food system, the increased diversity in crops, improved local value change chain, and diversified food system, result in increased household food security, improved livelihoods, and reduced hidden hunger, a virtuous cycle.<br>Policy-makers need to transform policy-making processes to represent the interests of different food system actors, promote indigenous crops, and support systems at all stages of the food system. This is an important point about the policy making process.	17
Limitations	25	The study had limited quantitative data to support some statistical analysis of the data.	

Conclusions	26	Our review highlights the unintended consequence of a commercialized food system, based on a reduced number of crops, as reinforcing of inequality and imbalances. While increasing national food security and stimulating national GDP, rather than making food cheaper and more accessible to all members of society, such a food system creates imbalances, reduces household food security, and exacerbates existing inequalities. Therefore, instead of improving the wellbeing of all, as envisaged in the Sustainable Development Goals, such a food system is disempowering, increases vulnerability of the most vulnerable, and creates legacy imbalances in outcomes such as health, wealth, and education, which will have long-term effects on national development and nation building. Such insights, explored in the South African context, have similar implications for other developing countries faced with competing policy agendas of increasing agricultural production for commercial growth and development, versus ensuring affordable and household food security for the most vulnerable citizenry, thereby decreasing social cohesion, and increasing the threat of social instability and conflict.	17
**FUNDING**			
Funding	27	This research was funded by the Wellcome Trust through the Sustainable and Healthy Food Systems (SHEFS) Project, the Water Research Commission of South Africa (WRC K5/2493//4), and the Adaptation Fund through the uMngeni Resilience Project. The APC was funded by the Wellcome Trust through the Sustainable and Healthy Food Systems (SHEFS) Project.	17

From: Moher D, Liberati A, Tetzlaff J, Altman DG, The PRISMA Group (2009). Preferred Reporting Items for Systematic Reviews and Meta-Analyses: The PRISMA Statement. PLoS Med 6(7): e1000097. doi:10.1371/journal.pmed1000097. For more information, visit: www.prisma-statement.org.
