# Supplementary material for: Mainstreaming Underutilized Indigenous and Traditional Crops into Food Systems: A South African Perspective
Source: Sustainability. Author manuscript; Available in PMC 2023 Sep 7. (PMC7615043; doi:10.3390/su11010172)
Supplement: PRISMA Flowchart [file EMS187259-supplement-PRISMA_Flowchart.pdf]

Figure S1: Process of article selection and number of articles selected for the review.

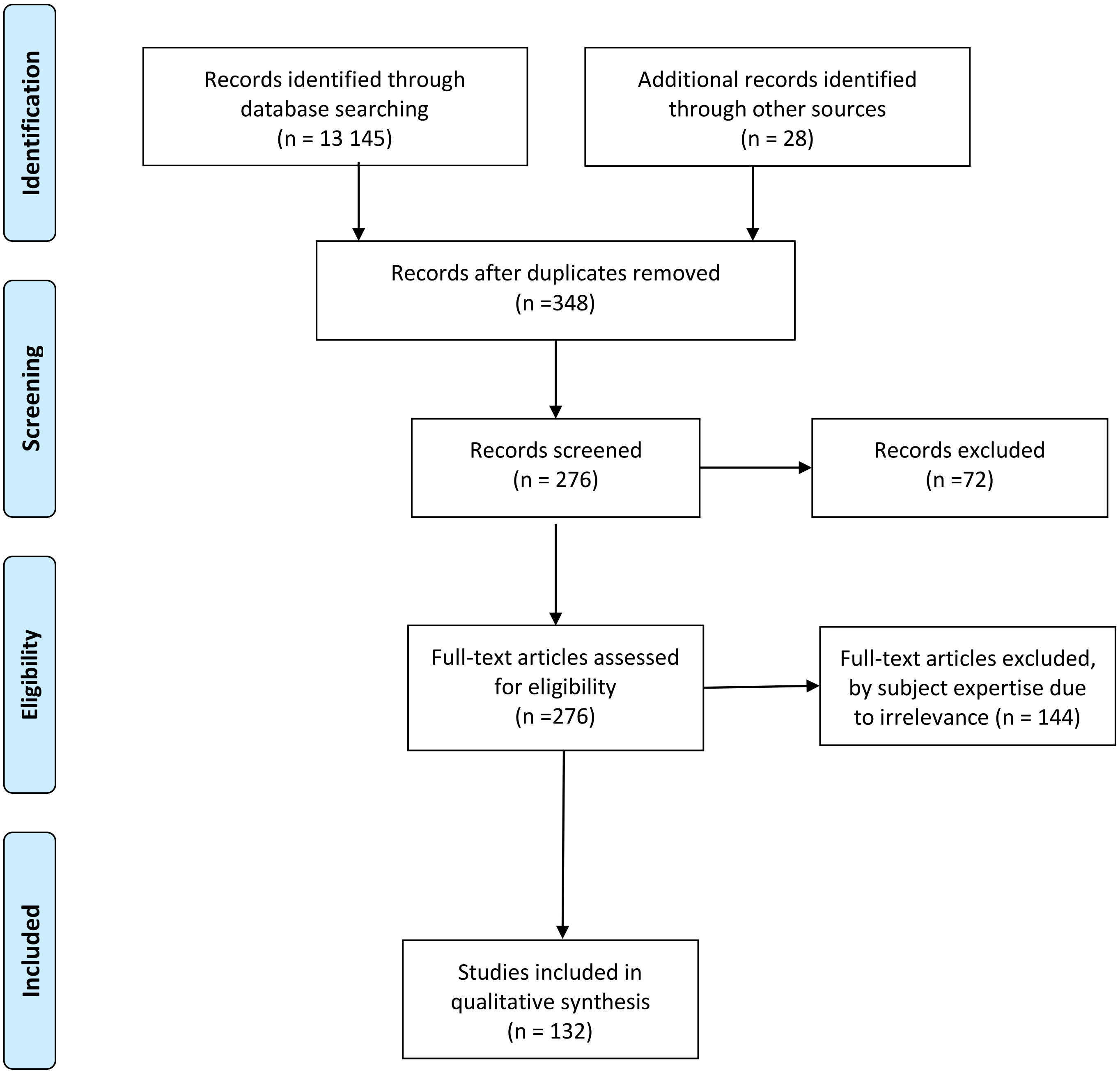

From: Moher D, Liberati A, Tetzlaff J, Altman DG, The PRISMA Group (2009). *P*referred *R*eporting *I*tems for *S*ystematic Reviews and *M*eta-*A*nalyses: The PRISMA Statement. PLoS Med 6(7): e1000097. doi:10.1371/journal.pmed1000097

For more information, visit www.prisma-statement.org.
